# A Novel Strategy for Decoding and Validating the Combination Principles of Huanglian Jiedu Decoction From Multi-Scale Perspective

**DOI:** 10.3389/fphar.2020.567088

**Published:** 2020-12-04

**Authors:** Ke-Xin Wang, Yao Gao, Wen-Xia Gong, Xiao-Feng Ye, Liu-Yi Fan, Chun Wang, Xue-Fei Gao, Li Gao, Guan-Hua Du, Xue-Mei Qin, Ai-Ping Lu, Dao-Gang Guan

**Affiliations:** ^1^ Modern Research Center for Traditional Chinese Medicine, Shanxi University, Taiyuan, China; ^2^ Institute of Integrated Bioinformedicine and Translational Science, Hong Kong Baptist University, Hong Kong, China; ^3^ Department of Orthopaedics and Traumatology, Nanfang Hospital, Southern Medical University, Guangzhou, China; ^4^ Institute of Basic Theory for Chinese Medicine, China Academy of Chinese Medical Sciences, Beijing, China; ^5^ Department of Physiology, School of Basic Medical Sciences, Southern Medical University, Guangzhou, China; ^6^ Institute of Materia Medica, Chinese Academy of Medical Sciences & Peking Union Medical College, Beijing, China; ^7^ Department of Biochemistry and Molecular Biology, School of Basic Medical Sciences, Southern Medical University, Guangzhou, China; ^8^ Guangdong Key Laboratory of Biochip Technology, Southern Medical University, Guangzhou, China

**Keywords:** system pharmacology, traditional Chinese medicine, compatibility, Dijkstra model, information graph algorithm

## Abstract

Traditional Chinese medicine (TCM) formulas treat complex diseases through combined botanical drugs which follow specific compatibility rules to reduce toxicity and increase efficiency. “Jun, Chen, Zuo and Shi” is one of most used compatibility rules in the combination of botanical drugs. However, due to the deficiency of traditional research methods, the quantified theoretical basis of herbal compatibility including principles of “Jun, Chen, Zuo and Shi” are still unclear. Network pharmacology is a new strategy based on system biology and multi-disciplines, which can systematically and comprehensively observe the intervention of drugs on disease networks, and is especially suitable for the research of TCM in the treatment of complex diseases. In this study, we systematically decoded the “Jun, Chen, Zuo and Shi” rules of Huanglian Jiedu Decoction (HJD) in the treatment of diseases for the first time. This interpretation method considered three levels of data. The data in the first level mainly depicts the characteristics of each component in single botanical drug of HJD, include the physical and chemical properties of component, ADME properties and functional enrichment analysis of component targets. The second level data is the characterization of component-target-protein (C-T-P) network in the whole protein-protein interaction (PPI) network, mainly include the characterization of degree and key communities in C-T-P network. The third level data is the characterization of intervention propagation properties of HJD in the treatment of different complex diseases, mainly include target coverage of pathogenic genes and propagation coefficient of intervention effect between target proteins and pathogenic genes. Finally, our method was validated by metabolic data, which could be used to detect the components absorbed into blood. This research shows the scientific basis of “Jun-Chen-Zuo-Shi” from a multi-dimensional perspective, and provides a good methodological reference for the subsequent interpretation of key components and speculation mechanism of the formula.

## Introduction

After thousands of years of experience in the treatment of diseases, the effectiveness of traditional Chinese medicine (TCM) is indisputable (Liang et al., 2015). During this process, TCM has also formed unique medication standards and compatibility theories, such as “Jun (emperor), Chen (minister), Zuo (adjuvant) and Shi (messenger)” (Wang et al., 2008; Lin and Yuhang, 2009). The basic and common feature of TCM is that it is consisted in the form of formula by the guidance of compatibility theory, different botanical drugs are orchestrated to form a multi-botanical drug combination of TCM (Lv et al., 2018). The principle of a formula is not just simply to mix botanical drugs, it is a process to increase efficiency and reduce toxicity. During this process, traditional compatibility rules will affect the changes of effective and toxic components of the formula, thus could reflect the advantages of enhancing synergistic effect and reducing toxicity of the formula (Liu et al., 2018). In TCM formula, botanical drugs are divided into “Jun, Chen, Zuo and Shi” according to the therapeutic characteristics of TCM, which are the basis of compatibility of TCM formula. “Jun” botanical drugs play leading roles in the treatment of diseases in the formula. “Chen” botanical drugs are usually worked as assistants to provide help to Jun botanical drugs in treating diseases; “Zuo” botanical drugs are used in combination with Jun and Chen botanical drugs to treat diseases or inhibit the toxic effects of Jun and Chen botanical drugs; “Shi” botanical drugs are widely used to coordinate the above botanical drugs and enhance their functions. It can be seen from this that the “Jun, Chen, Zuo and Shi” of the botanical drugs in the formula are mainly distinguished according to the primary and secondary roles of the medicine in the formula (Yao et al., 2013; Wu et al., 2014). Although “Jun, Chen, Zuo and Shi” is a widely used principle in the compatibility of TCM, the action pattern and underlying mechanisms of the compatibility rule of “Jun, Chen, Zuo and Shi” is still unclear. How to understand the action pattern and underlying mechanisms of compatibility rule is the basic and key step to decode the functional mechanisms of formulas in the treatment of complex diseases and benefit to secondary development of formulas.

A TCM formula generally contains several botanical drugs, and each botanical drug contains numbers of chemical components (Jia et al., 2004). These components are the material basis of the effect and mechanism of action (MOA) of the TCM formula. Studying the chemical composition and differences in efficacy of formula compatibility are helpful to clarify the pharmacological mechanism of TCM formula (Zhang, 2017). The traditional method to study the pharmacological effects of the single or several components in TCM is cell test or animal verify. These experiment-based research methods are divorced from the characteristics of the overall treatment of TCM, not only unable to tap the effective components of TCM, but also have no ability to effectively reveal the mechanism of TCM.

Network pharmacology has been widely used in the research of TCM in the treatment of complex diseases (Gu and Jianfeng, 2017). With the accumulation of omics data and the progress of network pharmacology technologies, increasing models are proposed to decode the therapeutic molecular mechanisms of formulas in treating complex diseases. For example, Wang et al. employed network pharmacology model combined with hypergeometric distribution to identify the enriched significant pathways. These pathways affected by a group of differentially expressed genes in pathway enrichment analysis, and further be used to reveal the mechanism of Wuwei-Ganlu-Yaoyu-Keli in treating rheumatoid arthritis (Wang et al., 2017). Gu et al. established a model for predicting signal transduction effects and extracting sub-networks by using EGS for mechanism analysis based on the data of TCM components and diseases (Gu et al., 2014). However, most models are used to decode the mechanism of TCM in treating complex diseases via networks analysis. Few models considered the compatibility rules and formulation principles of TCM, which are closely related to the effect and toxicity of drugs. He et al. studied the effect of compatibility of *Ginseng trifolium* (L.) Alph. Wood and *Aconitum carmichaeli* Debeaux on cardiac toxicity of rats through metabolomics and found that *Ginseng trifolium* (L.) Alph. Wood compatibility of *Aconitum carmichaeli* Debeaux can reduce its cardiac toxicity and increase its pharmacological effects by affecting the content of citric acid, glutathione, phosphatidyl choline and uric acid (He et al., 2015). In the treatment of rheumatoid arthritis, Zhang et al. observed that total glucosides of paeony combined with Tripteryginum wilfordii polyglycoside has better performance in the treatment of RA by increasing efficiency and reducing toxicity at the clinical applications (Zhang et al., 2019). How to utilize system pharmacology to analyze the principle of formulas in TCM at systemic level through mathematical and quantitative methods could be benefit for truly understanding the mechanisms of formula in the treatment of diseases.

Huanglian Jiedu Decoction (HJD) is composed of four botanical drugs, namely, *Coptis chinensis* Franch (Huanglian), *Scutellaria baicalensis* Georgi (Huangqin), *Phellodendron amurense* Rupr (Huangbo) and *Gardenia jasminoides* J. Ellis (Zhizi), with a compatible dosage of 3:2:2:3 (Ma et al., 2009). HJD is widely used in diabetes, cardiovascular and cerebrovascular diseases, inflammation, alzheimer’s disease, etc. in clinical applications (Wang and Xu, 2000; Xin et al., 2011; Zhang et al., 2014). It has a wide range of pharmacological activities such as antibacterial, anti-inflammatory, antioxidant, neuroprotective, etc (Lv et al., 2017). Previous pharmacological studies have shown that HJD could effectively control the weight of diabetic rats and has a good regulating effect on blood lipid and oxygen radical metabolism (Zhang et al., 2011). It has been reported that HJD has a strong lipid-regulating effect on type 2 diabetic rats, which can reduce the increase of pancreatic lipase activity in intestinal tract and inhibit the activity of pancreatic lipase *in vitro* (Zhang et al., 2014). In addition, pharmacological experimental study has found that the inflammatory factors in cerebrospinalfluid of AD rats demonstrated a callback trend after treatment with HJD, indicated that HJD can ameliorate the central inflammatory status of AD rats by regulating the levels of inflammatory factors (Gu et al., 2018). The above experimental results showed that HJD possessed obvious beneficial effects in the treatment of DM and AD.

Four botanical drugs in this formula are used for clearing away heat and toxic materials for thousands of years. In order to compare the effects of HJD and its botanical drugs on C. albicans biofilm formationin *in vitro*, Wang et al. found that the inhibitory effects of Huangqin, Huangbo on C. albicans biofilm were close to that of HJD, and Huanglian was superior to the other agents, Zhizi had no evidently inhibitory effect. Studies have also shown that HJD has obvious protective effect on cerebral ischemia injury (Wang et al., 2008). Wang et al. evaluated the effect of HJD and its botanical drugs on rabbit platelet aggregation and found that the aggregation inhibition rate of HJD was higher than that of each single botanical drug, and in four botanical drugs of HJD the aggregation inhibition rate of Huanglian was higher than that of the other three botanical drugs (Wang et al., 2014). The above applications of “Jun, Chen, Zuo and Shi” of HJD show that the relationship among “Jun, Chen, Zuo and Shi” does exists and has quantitative basis in modern pharmacology and experimental scientific research. How to detect the action pattern and underlying mechanisms of “Jun, Chen, Zuo and Shi” at a systematic and global perspective is the key to understand the mechanisms of formulas in the treatment of complex diseases in TCM.

In this study, the compatibility rules of “Jun, Chen, Zuo and Shi” of HJD in the treatment of diseases are systematically interpreted. Specifically, our new system pharmacology strategy integrated three levels of data including the characteristics of each component in HJD single botanical drug, the characterization of component-target-protein (C-T-P) network in the whole protein-protein interaction (PPI) network and the characterization of intervention propagation properties of HJD in the treatment of different complex diseases. Overall, our study provides a comprehensive systems pharmacology framework to decode the principles of “Jun-Chen-Zuo-Shi” from multi-level perspective, which may give some enlightenment for the subsequent interpretation of key components and hidden mechanism of the formula.

## Materials and Methods

### Chemical Components Collection

All chemical components of HJD and seven important pharmacological related descriptors (MW, ALOGP, HDON, HACC, CACO-2, OB (%) and DL) for each component were collected from Traditional Chinese Medicine Systems Pharmacology (TCMSP) database (Ru et al., 2014) (http://lsp.nwsuaf.edu.cn/tcmsp.php). The chemical identification and concentration of in HJD were collected from the previous reports (Yang et al., 2019). All chemical structures were prepared and converted into canonical SMILES using Open Babel Toolkit (version 2.4.1). The targets of HJD were predicted by using Similarity Ensemble Approach SEA (Keiser et al., 2007) (http://sea.bkslab.org/), hitpick (Liu et al., 2013) (http://mips.helmholtz-muenchen.de/proj/hitpick) and Swiss Target Prediction (David et al., 2014) (http://www.swisstargetprediction.ch/).

### Active Components Screening

ADME properties of drugs refer to the Absorption, Distribution, Metabolism and Elimination, which are the key properties of whether small molecules of drugs can be used as medicines. It is estimated that due to low intestinal absorption rate and poor metabolic stability, the oral bioavailability of drugs is low, and finally about 50% of drugs fail in clinical trials (Beaumont et al., 2014). Therefore, ADME predictive screening of drugs is particularly important in drug discovery.

Oral bioavailability (OB) refers to the percentage of oral dose of drugs reaching the blood circulation system, which is the most common pharmacokinetic parameter for drug screening (Chiou, 2001). For specific oral drugs, due to poor intestinal absorption, drug metabolism, efflux and other reasons, the number of drugs that eventually reach the circulatory system is greatly reduced, so the oral availability of drugs ranges from 0 to 100%. As the initial absorption rates of intestinal tract and liver are generally about 43 and 44% respectively, components with OB greater than or equal to 30% are selected as active components.

Drug-like (DL) refers to a class of components that have the same functional group or similar physical characteristics as most known drugs (Han et al., 2011). The drug-like index of a new component is calculated based on Tanimoto similarity. According to the drug-like index, molecules with drug-like properties less than 0.14 are eliminated. Finally, the components screened according to OB and drug-like index take intersection as the active component (Wang et al., 2018).

### Network Construction

The C-T-P network of HJD were constructed by using Cytoscape wsoftware (Version 3.7.0) (Lopes et al., 2010). The networks and topological parameters were analyzed using NetworkAnalyzer, which is a plugin of Cytoscape (de Jong et al., 2003). The PPI data were derived from public databases BioGRID, STRING, Dip, HPRD, Intact, Mint and Reactome (Guan et al., 2014).

### Detection of Functional Communities Structure in HJD

Community structures in the biomedical network are more important for annotating the biological means. One index *codebook* and *n community codebooks* were defined to character the movements of random walker within and between communities respectively. Community codebook *x* has one codeword for each node *α∈x* and one exit codeword. The frequency at which random walkers visit each node in the community is the codeword length, *k*_*α∈x*_, and exits the community, $s_{x↷}$. The was used to denote the sum of these frequencies, the total use of codewords in community *x*, and *K* ^*x*^ to denote the normalized probability distribution. Consistent with the above, the index codebook has the community entries of codewords. The codeword lengths are derived from the set of frequencies at which the random walker enters each community. The was used to denote the sum of these frequencies, the total use of codewords to move into communities, and *S* to denote the normalized probability distribution. We want to express average length of codewords from the index codebook and the community codebooks weighted by their rates of use. Therefore, the map equation is

$$L\left(\text{N}\right)=s_{↶}H\left(S\right)+\overset{n}{\underset{x=1}{\sum }}k_{x↻}H\left(k_{x}\right)$$ (1)

Next, we elaborate on the terms of the map equation in detail and illustrate it with Hoffman code examples.

*L*(N) represents the per-step description length for community partition *N*. That is, for community partition *N* of n nodes into *n* communities, the lower bound of the average length of the code describing a step of the random walker.

$$s_{↶}=\overset{n}{\underset{x=1}{\sum }}s_{x↶}$$ (2)

The index codebook rate is used. The per-step use rate of the index codebook is given by the total probability that the random walker enters any of them communities. This variable represents the proportion of all codes representing community names in the codes. Where is probability of jumping out of community *x*.

$$\;\;\;H\left(S\right)=-\overset{n}{\underset{x=1}{\sum }}\left(s_{↶}/s_{x↶}\right)\text{log}\left(s_{x↶}/s_{↶}\right)$$ (3)

This variable represents the average byte length required to encode community names. The frequency-weighted average length of codewords in the index codebook. The entropy of the relative rates to use the motif codebooks measures the smallest average codeword length that is theoretically possible. The heights of individual blocks under *Index codebook* correspond to the relative rates and the codeword lengths approximately correspond to the negative logarithm of the rates in base 2.

$$k_{x↻}=\underset{\alpha \in x}{\sum }k_{\alpha }+s_{x↷}$$ (4)

This variable represents the coding proportion of all nodes (including jump-out nodes) belonging to community *x* in the coding. The rate at which the community codebook *x* is used, which is given by the total probability that any node in the community is visited, plus the probability that the random walker exits the community and the exit codeword is used.

$$H\left(k^{x}\right)=-\left(s_{x↷}/k_{x↻}\right)\text{log}\left(s_{x↷}/k_{x↻}\right)-\underset{\alpha \in x}{\sum }\left(k_{a}/k_{x↻}\right)\text{log}\left(k_{a}/k_{x↻}\right)$$ (5)

This variable represents the average byte length required to encode all nodes in community *x*. The frequency-weighted average length of codewords in community codebook *x*. The entropy of the relative rates at which the random walker exits community *x* and visits each node in community *x* measures the smallest average codeword length that is theoretically possible. The heights of individual blocks under community codebooks correspond to the relative rates and the codeword lengths approximately correspond to the negative logarithm of the rates in base 2.

### Propagation Coefficient Calculation

The role of drug response in the body is a complex process involving different proteins or genes. However, these proteins and genes are regulated by different cellular components, which constitute complex network relationships in the process of disease occurrence and development. These network relations could propagate the therapeutic effects through the network and orchestrate the cascade path of drug response. At present, most network pharmacological models just focus on the direct relationships between drugs and targets, and do not consider the propagation mode and propagation effect of drug intervention. In this study, we use Dijkstra model to detect the shortest distance from the direct target to the pathogenic gene based on C-T-P network, and keep the path with less than or equal to three nodes in the shortest distance. We believe that the initial node is the direct target of components, the final node is the pathogenic gene, which is also defined as the number of reached effector proteins, and the intermediate node is defined as the mode of propagation. Components with more modes of propagation and more effector proteins usually have better intervention effects. Based on the number of reached effector proteins and the mode of propagation, we calculated the propagation coefficient of each single botanical drug in HJD.

We defined the C-T-P network as the graph G (V, E), V and E represents nodes and edges, respectively (u, v) represents edge in E, W_*u,v*_ stand for the weight of the edge. V is divided into two sets S and T, where the distance from the targets in S to U has been determined and the distance from the target to U contained in T has not been determined. Then, the distance from u to the target x in T is set as d_*u,x*_, which is defined as the shortest path length from u to the target x in T. The specific formula is as follows:

 1) At the beginning, S = {u}, T = V-{u}. For all targets x in t, if there is a path from u to x, then d_*u,x*_ = *w*_*u,v*_; Otherwise, set d_*u,x*_ = ∞.
 2) For all targets x in T, find the target T with smallest d_*u,x*_, i.e.:
 d_*u,t*_ = min{d_*u,x*_∣x∈T∣}
 d_*u,t*_ is the shortest distance from target t to effector proteins u. At the same time, target T is also the target closest to u among all targets in set T. Delete target t from T and merge it into S.
 3) For all targets x adjacent to t in T, update the values of d_*u,x*_ with the following formula:
 d_*u,x*_ = min{d_*u,x*_, d_*u,t*_ + *w*_*t,x*_}
 4) Continue the above steps until T is an empty set.
 5) During calculation of d_*u,x*_ holding paths less than or equal to 3 nodes as the shortest distance dmin, in a path with three nodes, the first node mean components targets, the second node represent propagation modes, the third node represent effector proteins, the formula for calculating the propagation coefficient of each single botanical drug is:

$${\mathrm{PC}}_{\mathrm{(botanical drug)}}=\frac{\sum_{i}^{n}dmin/n}{\sum_{j}^{m}u/m}$$ (6)

The propagation coefficient (PC) represents the strength of intervention ability of a botanical drug. The n represents the number of propagation modes, the m represents the number of effector proteins.

### KEGG Pathway

To analyze the main function of botanical drugs in HJD, the latest pathway data were obtained from the Kyoto Encyclopedia of Genes and Genomes (KEGG) database (Draghici et al., 2007) were extracted for KEGG pathway enrichment analyses. *p*-values were set at 0.05 as the cut-off criterion. The results of analysis were annotated by Pathview (Luo and Brouwer, 2013) in the R Bioconductor package (https://www.bioconductor.org/).

### Statistical Analysis

To compare the importance of communities in HJD, SPSS22.0 was used for statistical analysis. One-way analysis of variance followed by a Dunnett post-hoc test was used to compare more than two groups. Obtained *p*-values were corrected by Benjamini-Hochberg false discovery rate (FDR). Results were considered as statistically significant if the *p*-value was <0.05.

## Results

**FIGURE 1:**
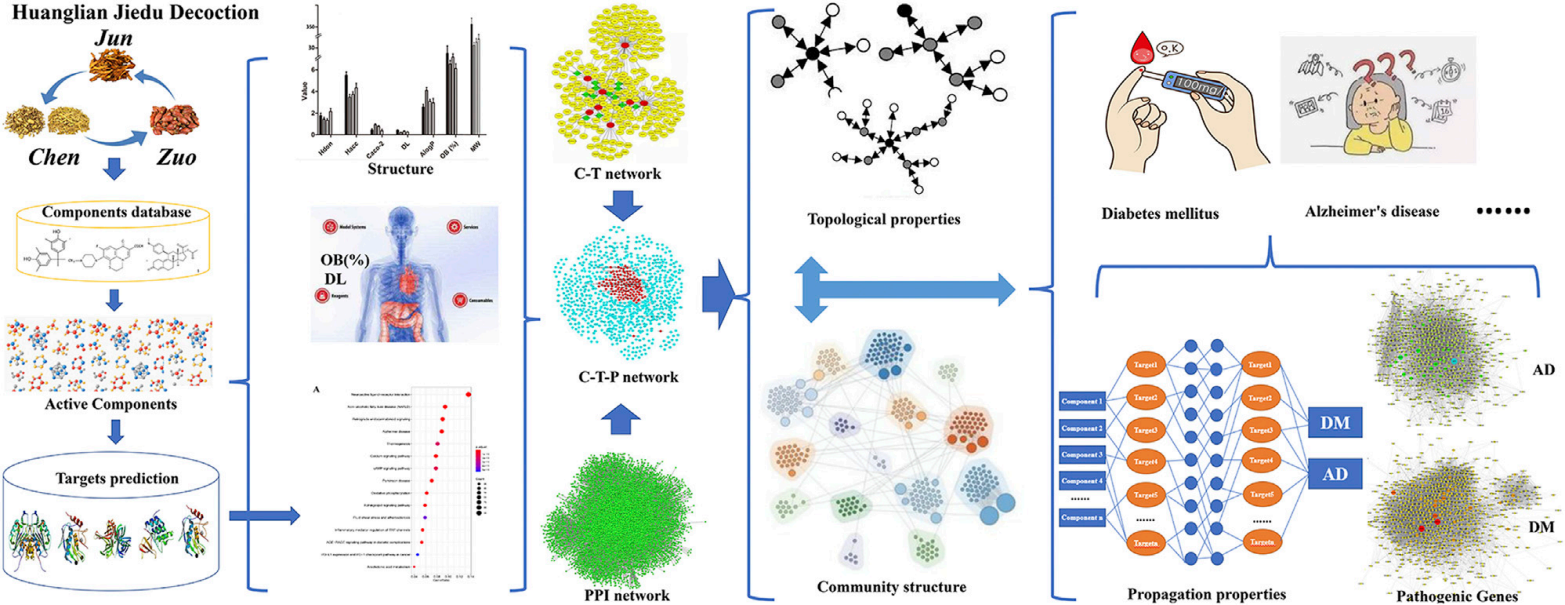
A schematic diagram of network pharmacology-based strategy to decode the principles of “Jun-Chen-Zuo-Shi” in TCM.

“Jun-Chen-Zuo-Shi” is one of the widely used compatibility principles of TCM in the treatment of diseases. However, there is still lack researches on the compatibility principle of “Jun-Chen-Zuo-Shi”. In this study, we systematically analyze the compatibility principle and mechanism of HJD based on “Jun-Chen-Zuo-Shi” at three levels: the characteristics of each component in single botanical drug of HJD, including the physical and chemical properties of component, ADME properties and functional enrichment analysis of component targets; the characterization of C-T-P network in the whole PPI network, mainly including the characterization of degree and key communities in C-T-P network; the characterization of intervention propagation properties of HJD in the treatment of different complex diseases, mainly including target coverage of pathogenic genes and propagation coefficient of intervention effect between targets and pathogenic genes (Figure 1).

### Comparison of Chemical Properties of Components

**FIGURE 2:**
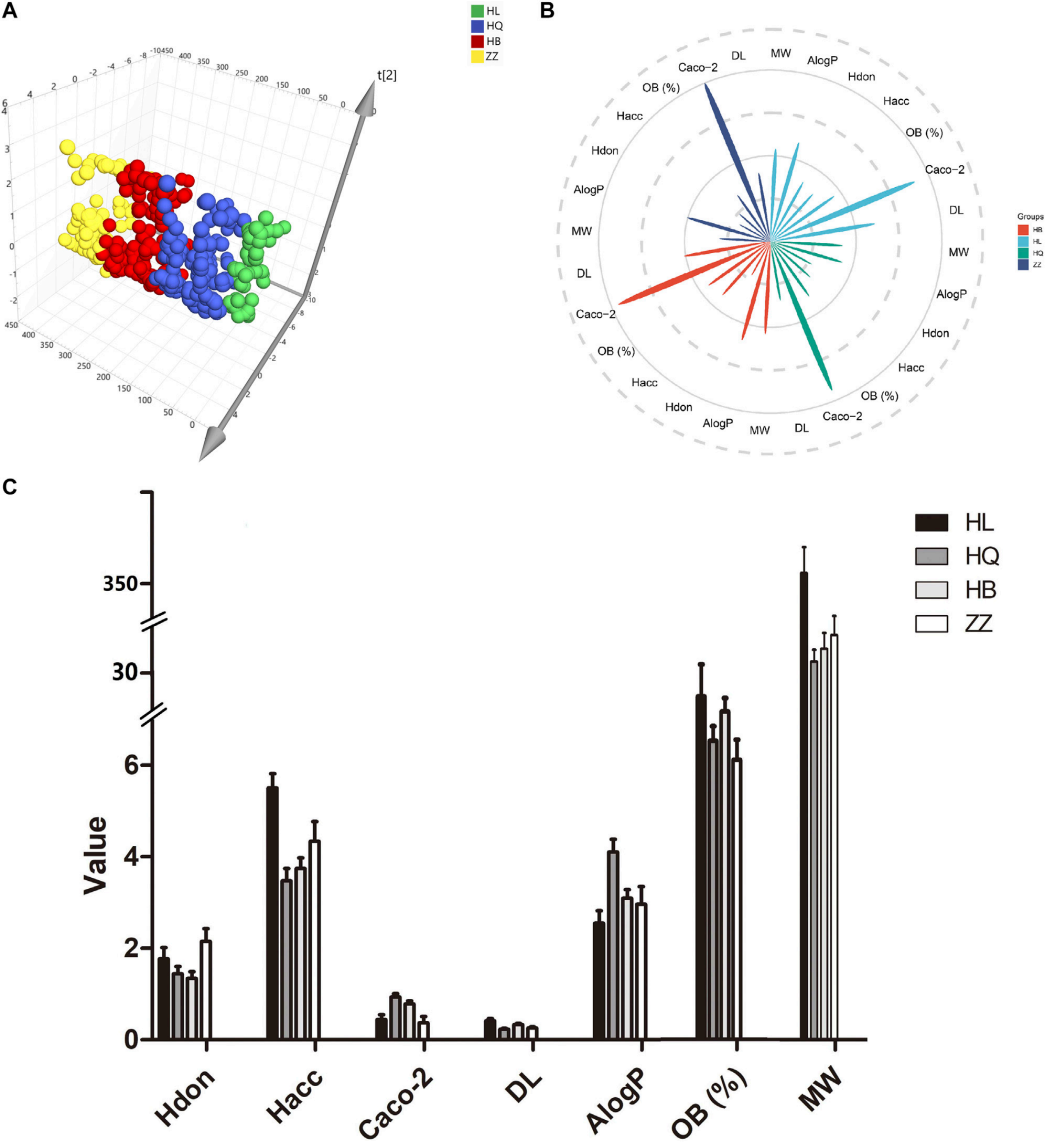
Analysis seven chemical properties of huanglian (HL), huangqin (HQ), huangbo (HB) and zhizi (ZZ) in HJD. **A**, **B** and **C** represent the chemical space, chemical distribution and the value of chemical parameters of all components visualized by PCA scores plot, polar coordinate petal diagram and bar chart, respectively.

Physical and chemical properties of drugs directly affect the activity of drugs and play important roles in the druggability of TCM. In order to detect the importance of these physical and chemical properties in “Jun-Chen-Zuo-Shi”, seven important pharmacologically related descriptors, MW, ALOGP, HDON, HACC, CACO-2, OB (%) and DL, were analyzed from four botanical drugs in HJD. Principal component analysis (PCA) widely used as a pattern recognition method to reflect the most primitive state of data in an unsupervised state. Here, PCA was applied to detect the distribution of the above physical and chemical properties of four botanical drugs in “Jun-Chen-Zuo-Shi”. The results showed that Huangqin and Huangbo are closest to Huanglian, indicating that their physical and chemical properties are close to Huanglian. Zhizi is farthest from Huanglian, which indicates that their physical and chemical properties are quite different in PCA scatter plot (Figure 2A). To further describe the specific differences among the four botanical drugs, we have made a detailed analysis of the seven parameters. As shown in Figures 2B,C, 1) For MW, the average value of all components in huanglian (342.2) is higher than that of huangqin (277.7), huangbo (287) and zhizi (297). 2) For bioavailability, the average OB value (%) of huanglian (36.09) is also higher than that of huangqin (31.42), huangbo (34.51) and zhizi (29.43). (3) For permeability, the average Caco-2 value of huanglian (0.4423) is lower than that of huangqin (0.9371) and huangbo (0.7851). (4) For DL, like MW and OB, huanglian possessed higher average DL value (0.4165), that is very different from that of huangqin (0.2301), huangbo (0.3303) and zhizi (0.258). (5) Compared with the all components of huanglian (2.551), the ALogP value of huangqin (4.101), huangbo (3.096) and zhizi (2.964) exhibited siginifically higher average ALogP values, which indicates the majority components in huangqin, huangbo and zhizi are hydrotropic, but that in huanglian are hydrophobic. (6) The values of nHAcc in huanglian (5.5) are all higher than those in others (3.476, 3.743, 4.337, respectively). The above analysis results show that the chemical properties of the four botanical drugs are obviously different. Therefore, we can speculate that each botanical drug plays a different role in the compatibility of this TCM. The Jun botanical drug huanglian is distinguished based on the compatibility principle of “Jun-Chen-Zuo-Shi”, and has good performance in most physical and chemical properties such as OB (%), DL and MW, which indicated that it may play a leading role at the functional level.

### Chemical Analysis in HJD

Chemical analysis plays an important role in the study of the substances basis and mechanism of botanical drugs in the formula. The information on specific chemical identification and concentration of the botanical drugs in HJD were collected by searching from the literature (Yang et al., 2019). The detailed information was shown in Table 1. The results suggest that the chemical components of botanical drugs and the concentration of identified components provide an experiment-aided chemical space for the search of active components. This will provide valuable reference for further analysis.

**TABLE 1:** The information on chemical analysis of the botanical drugs from the literature in HJD.

Formula	Method	Component	Concentration (mg/g)	Ref
Huanglian Jiedu decoction (HJD)	HPLC	Phellodendrine	3.8276 ± 0.1158	(Yang et al., 2019)
		Heriguard	1.0800 ± 0.0261	
		Magnoflorine	6.7489 ± 0.0450	
		Geniposide	72.3830 ± 1.0948	
		Coptisine	14.0580 ± 0.1631	
		Epiberberine	8.9056 ± 0.0864	
		Jatrorrizine	9.4028 ± 0.0966	
		Berberine	53.0820 ± 0.5443	
		Palmatine	19.6820 ± 0.1452	
		Baicalin	18.5770 ± 0.0927	
		Oroxindin	17.5360 ± 0.2370	
		Wogonin	2.1689 ± 0.3488	
		Oroxylin A	0.2618 ± 0.0212	

### Comparison of Active Components

TCM formula contains a large number of chemical molecules, the traditional methods of exploring the active components in TCM are mainly based on the separation, purification and structure analysis of mass spectrometry (MS) and high-performance liquid chromatography (HPLC). These methods supplied quantitative concentration of components, however, these experiment-based methods cost a lot of manpower, material and financial resources to excavate the effective components in TCM. Therefore, it is particularly important to analyze, explore and optimize the TCM formula by calculating ADME properties, which could be helpful to screen the potential active components of TCM, and further optimize the TCM formula, improve the research and development of new drugs from TCM. In this study, botanical drug components were evaluated by using the two representative ADME parameters, oral bioavailability (OB) and drug-like (DL), to screen the active components of TCM formula.

Our statistic results show that 26.81% (94) of the components in HJD meet OB ≥ 30% and DL ≥ 0.14 (Table 2). Specifically, 31.25% of the components in Huanglian satisfy OB ≥ 30% and DL ≥ 0.14. These components are regarded as the active components in Huanglian, which included berberine, coptisine, epiberberine, and palmatine, etc. Studies have shown that berberine could significantly reduce hyperglycemia and glycogen content in liver of diabetic mice, increase the expression of Akt and IRS, and inhibit the expression of GSK-3β (Xie et al., 2011). It has been reported that berberine can reduce the release of neuroamyloid through PI3K/Akt/GSK3 pathway, decrease the number of senile plaques in the brain of AD mice model, and play a therapeutic role in AD (Durairajan et al., 2012). Zhai et al. has reported that coptisine could improve oxidative renal injury in diabetic rats, and the potential mechanisms may be associated to activation of the Nrf2 signaling pathway (Zhai et al., 2019). Jung et al. found that epiberberine has a strong potential of inhibition and prevention of AD mainly through ChEs and beta-amyloids pathways, and additionally through antioxidant capacities (Jung et al., 2009). Previous pharmacological studies have shown that palmatine treatment can alleviate the hyperalgesia, allodynia and depressive behaviors of rats with comorbidity of diabetic neuropathic pain and depression (Shen et al., 2018). 27.27% of the components in Huangqin meet OB ≥ 30% and DL ≥ 0.14, including most common active components such as coptisine, epiberberine, wogonin, and oroxylin A, etc. Khan et al. (2016) found that wogonin administration could suppress hyperglycemia, improve cardiac function, and mitigate cardiac fibrosis in STZ-induced diabetic mice. 29.28% of the molecules in Huangbo meet OB ≥ 30% and DL ≥ 0.14, a total of 41 components meet the threshold selection criteria, such as coptisine, berberine, and palmatine, etc. Only 20.41% of the molecules in Zhizi meet OB ≥ 30% and DL ≥ 0.14, including oleic acid, kaempferol and mandenol, etc. The above results suggest that the number of active components retained by Huanglian are the highest compared with those before screening. This shows that more components in Huanglian have better OB and DL properties, and indicates that these components may play a major therapeutic role in the treatment disease. Huangqin and Huangbo have the second highest proportion of active components, and have more overlapping components with Huanglian, which intimates that Huangqin and Huangbo can assist and enhance therapeutic effect of Huanglian. In HJD, Zhizi is the Zuo botanical drug with the lowest content of active components, indicating that it may have auxiliary effect on Jun botanical drugs and/or Chen botanical drugs.

**TABLE 2:** Components in HJD for further analysis after ADME screening.

No	Component	OB (%)	DL	botanical drug		No	Component	OB (%)	DL	botanical drug	
HJD1	Quercetin	46.43	0.28	Huanglian	Jun	HJD58	Magnograndiolide	63.71	0.19	Huangbo	Chen
HJD2	Magnograndiolide	63.71	0.19	Huanglian	Jun	HJD59	Oleic acid	33.13	0.14	Huangbo	Chen
HJD3	Palmidin A	35.36	0.65	Huanglian	Jun	HJD60	Palmidin A	35.36	0.65	Huangbo	Chen
HJD4	Corchoroside A_qt	104.95	0.78	Huanglian	Jun	HJD61	phellamurin_qt	56.6	0.39	Huangbo	Chen
HJD5	Obacunone	43.29	0.77	Huanglian	Jun	HJD62	Poriferast-5-en-3beta-ol	36.91	0.75	Huangbo	Chen
HJD6	Palmatine	64.6	0.65	Huanglian	Jun	HJD63	Kihadalactone A	34.21	0.82	Huangbo	Chen
HJD7	Berberine	36.86	0.78	Huanglian	Jun	HJD64	Phellavin_qt	35.86	0.44	Huangbo	Chen
HJD8	Coptisine	30.67	0.86	Huanglian	Jun	HJD65	Delta 7-stigmastenol	37.42	0.75	Huangbo	Chen
HJD9	Fagarine	72.23	0.15	Huanglian	Jun	HJD66	Phellopterin	40.19	0.28	Huangbo	Chen
HJD10	Worenine	45.83	0.87	Huanglian	Jun	HJD67	Dehydrotanshinone II A	43.76	0.4	Huangbo	Chen
HJD11	Berberrubine	35.74	0.73	Huanglian	Jun	HJD68	Dihydroniloticin	36.43	0.81	Huangbo	Chen
HJD12	Epiberberine	43.09	0.78	Huanglian	Jun	HJD69	Kihadanin A	31.6	0.7	Huangbo	Chen
HJD13	(R)-Canadine	55.37	0.77	Huanglian	Jun	HJD70	Niloticin	41.41	0.82	Huangbo	Chen
HJD14	Berlambine	36.68	0.82	Huanglian	Jun	HJD71	Chelerythrine	34.18	0.78	Huangbo	Chen
HJD15	Moupinamide	86.71	0.26	Huanglian	Jun	HJD72	Candletoxin A	31.81	0.69	Huangbo	Chen
HJD16	Ent-Epicatechin	48.96	0.24	Huangqin	Chen	HJD73	Hericenone H	39	0.63	Huangbo	Chen
HJD17	EIC	41.9	0.14	Huangqin	Chen	HJD74	Hispidone	36.18	0.83	Huangbo	Chen
HJD18	Wogonin	30.68	0.23	Huangqin	Chen	HJD75	Campesterol	37.58	0.71	Huangbo	Chen
HJD19	(2 R)-7-hydroxy-5-methoxy-2-phenylchroman-4-one	55.23	0.2	Huangqin	Chen	HJD76	Melianone	40.53	0.78	Huangbo	Chen
HJD20	Beta-sitosterol	36.91	0.75	Huangqin	Chen	HJD77	Phellochin	35.41	0.82	Huangbo	Chen
HJD21	Sitosterol	36.91	0.75	Huangqin	Chen	HJD78	Obacunone	43.29	0.77	Huangbo	Chen
HJD22	Stigmasterol	43.83	0.76	Huangqin	Chen	HJD79	Palmatine	64.6	0.65	Huangbo	Chen
HJD23	Norwogonin	39.4	0.21	Huangqin	Chen	HJD80	Fumarine	59.26	0.83	Huangbo	Chen
HJD24	5,2′-dihydroxy-6,7,8-trimethoxyflavone	31.71	0.35	Huangqin	Chen	HJD81	Isocorypalmine	35.77	0.59	Huangbo	Chen
HJD25	Coptisine	30.67	0.86	Huangqin	Chen	HJD82	Berberine	36.86	0.78	Huangbo	Chen
HJD26	Supraene	33.55	0.42	Huangqin	Chen	HJD83	(S)-Canadine	53.83	0.77	Huangbo	Chen
HJD27	Acacetin	34.97	0.24	Huangqin	Chen	HJD84	Coptisine	30.67	0.86	Huangbo	Chen
HJD28	Methyl linolelaidate	41.93	0.17	Huangqin	Chen	HJD85	N-Methylflindersine	32.36	0.18	Huangbo	Chen
HJD29	Baicalein	33.52	0.21	Huangqin	Chen	HJD86	delta7-dehydrosophoramine	54.45	0.25	Huangbo	Chen
HJD30	Diop	43.59	0.39	Huangqin	Chen	HJD87	Rutaecarpine	40.3	0.6	Huangbo	Chen
HJD31	Epiberberine	43.09	0.78	Huangqin	Chen	HJD88	Skimmianin	40.14	0.2	Huangbo	Chen
HJD32	5,7,2,5-Tetrahydroxy-8,6-dimethoxyflavone	33.82	0.45	Huangqin	Chen	HJD89	Fagarine	72.23	0.15	Huangbo	Chen
HJD33	Carthamidin	41.15	0.24	Huangqin	Chen	HJD90	Worenine	45.83	0.87	Huangbo	Chen
HJD34	2,6,2′,4′-tetrahydroxy-6′-methoxychaleone	69.04	0.22	Huangqin	Chen	HJD91	Cavidine	35.64	0.81	Huangbo	Chen
HJD35	Dihydrobaicalin_qt	40.04	0.21	Huangqin	Chen	HJD92	Berberrubine	35.74	0.73	Huangbo	Chen
HJD36	Eriodyctiol (flavanone)	41.35	0.24	Huangqin	Chen	HJD93	Ptelein	72.44	0.15	Huangbo	Chen
HJD37	Salvigenin	49.07	0.33	Huangqin	Chen	HJD94	Thalifendine	44.41	0.73	Huangbo	Chen
HJD38	5,2′,6′-trihydroxy-7,8-dimethoxyflavone	45.05	0.33	Huangqin	Chen	HJD95	Quercetin	46.43	0.28	Zhizi	Zuo
HJD39	5,7,2′,6′-tetrahydroxyflavone	37.01	0.24	Huangqin	Chen	HJD96	EIC	41.9	0.14	Zhizi	Zuo
HJD40	Dihydrooroxylin A	38.72	0.23	Huangqin	Chen	HJD97	Beta-sitosterol	36.91	0.75	Zhizi	Zuo
HJD41	Skullcapflavone II	69.51	0.44	Huangqin	Chen	HJD98	Kaempferol	41.88	0.24	Zhizi	Zuo
HJD42	Oroxylin a	41.37	0.23	Huangqin	Chen	HJD99	Stigmasterol	43.83	0.76	Zhizi	Zuo
HJD43	Panicolin	76.26	0.29	Huangqin	Chen	HJD100	Oleic acid	33.13	0.14	Zhizi	Zuo
HJD44	5,7,4′-trihydroxy-8-methoxyflavone	36.56	0.27	Huangqin	Chen	HJD101	Crocetin	35.3	0.26	Zhizi	Zuo
HJD45	NEOBAICALEIN	104.34	0.44	Huangqin	Chen	HJD102	Mandenol	42	0.19	Zhizi	Zuo
HJD46	DIHYDROOROXYLIN	66.06	0.23	Huangqin	Chen	HJD103	Supraene	33.55	0.42	Zhizi	Zuo
HJD47	Moslosooflavone	44.09	0.25	Huangqin	Chen	HJD104	METHYL LINOLEATE	41.93	0.17	Zhizi	Zuo
HJD48	11,13-Eicosadienoic acid, methyl ester	39.28	0.23	Huangqin	Chen	HJD105	(4aS,6 aR,6aS,6bR,8 aR,10R,12 aR,14bS)-10-hydroxy-2,2,6a,6b,9,9,12a-heptamethyl-1,3,4,5,6,6a,7,8,8a,10,11,12,13,14 b-tetradecahydropicene-4a-carboxylic acid	32.03	0.76	Zhizi	Zuo
HJD49	Linolenic acid methyl ester	46.15	0.17	Huangqin	Chen	HJD106	Methyl vaccenate	31.9	0.17	Zhizi	Zuo
HJD50	5,7,4′-trihydroxy-6-methoxyflavanone	36.63	0.27	Huangqin	Chen	HJD107	Ammidin	34.55	0.22	Zhizi	Zuo
HJD51	5,7,4′-trihydroxy-8-methoxyflavanone	74.24	0.26	Huangqin	Chen	HJD108	Isoimperatorin	45.46	0.23	Zhizi	Zuo
HJD52	Rivularin	37.94	0.37	Huangqin	Chen	HJD109	Exceparl M-OL	31.9	0.16	Zhizi	Zuo
HJD53	bis [(2 S)-2-ethylhexyl] benzene-1,2-dicarboxylate	43.59	0.35	Huangqin	Chen	HJD110	Ethyl oleate (NF)	32.4	0.19	Zhizi	Zuo
HJD54	5,8,2′-trihydroxy-7-methoxyflavone	37.01	0.27	Huangqin	Chen	HJD111	5-Hydroxy-7-methoxy-2-(3,4,5-trimethoxyphenyl)chromone	51.96	0.41	Zhizi	Zuo
HJD55	Quercetin	46.43	0.28	Huangbo	Chen	HJD112	3-Methylkempferol	60.16	0.26	Zhizi	Zuo
HJD56	Beta-sitosterol	36.91	0.75	Huangbo	Chen	HJD113	GBGB	45.58	0.83	Zhizi	Zuo
HJD57	Stigmasterol	43.83	0.76	Huangbo	Chen	HJD114	Sudan III	84.07	0.59	Zhizi	Zuo

### Coverage Rate Based on Functional Pathway

**FIGURE 3:**
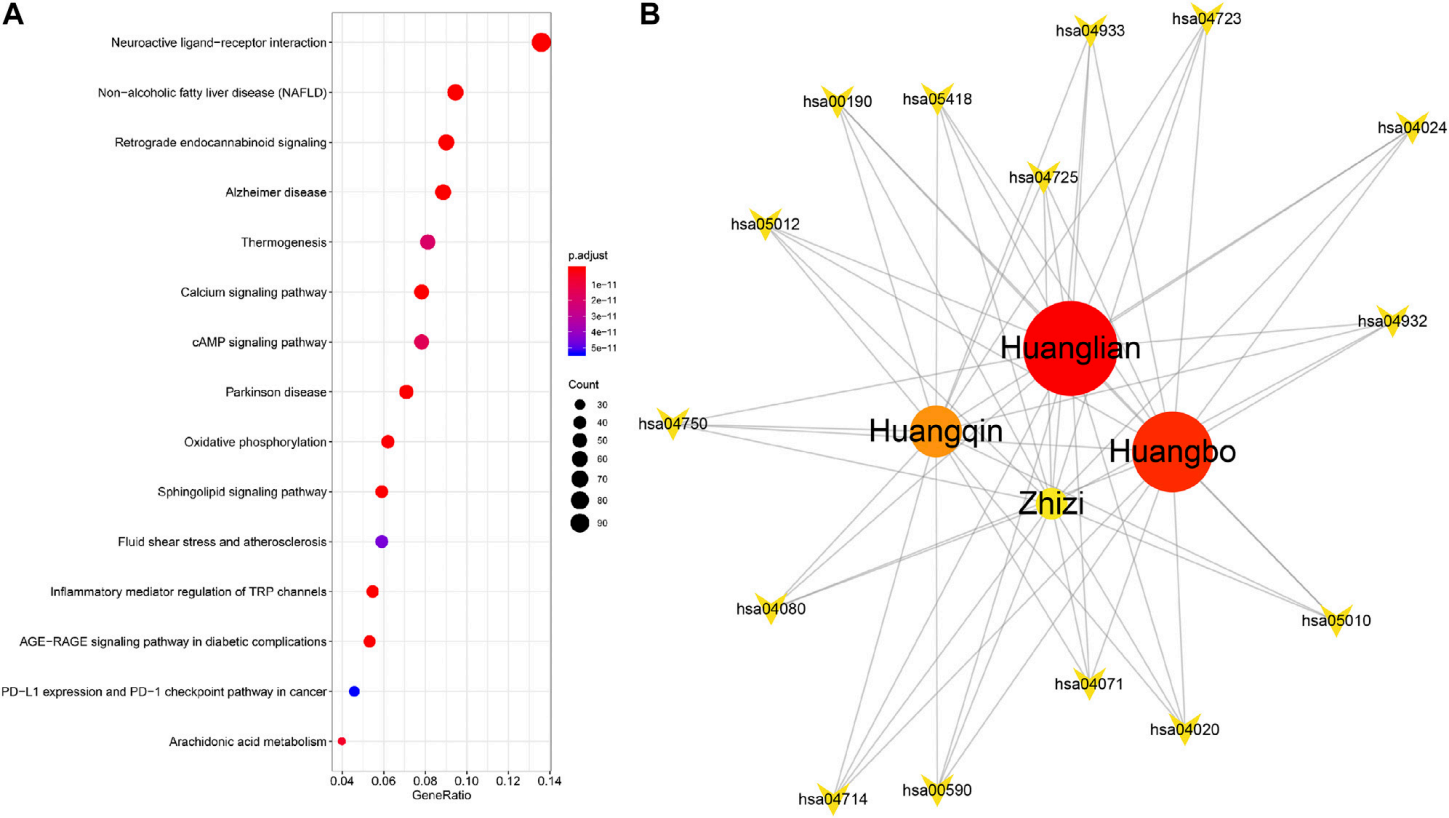
Gene enrichment analysis of all targets from HJD **(A)**. botanical drug-pathway network of HJD **(B)**. The circle nodes represent botanical drugs, and the inverted triangle represents the top 15 pathways of HJD. The size and color of the node represents the importance of the herbal regulation pathway.

Most complex diseases are not caused by a single pathological change, but a series of physiological reactions caused by abnormal pathways due to disorder of multiple proteins or genes in the cell. In order to further explore the “Jun-Chen-Zuo-Shi” compatibility principle of HJD at the potential molecular mechanism level, we evaluated it through the functional pathway enrichment analysis based on KEGG (Figure 3A). Previous reports confirm that HJD has significant therapeutic effects on Alzheimer’s disease (AD), Parkinson’s disease (PD) and diabetes mellitus (DM) etc. The top 15 enriched pathways were selected for further analysis. The targets of each botanical drug in HJD was mapped to the enriched genes involved in these 15 enriched pathways for enrichment analysis. It was found that 40.54, 33.14, 38.10, and 29.69% of the targets in the Jun (Huanglian), Chen (Huangqin and Huangbo) and Zuo (Zhizi) botanical drug were enriched in the top 15 enriched pathways, respectively (Figure 3B). The above analysis show that Huanglian has a higher target contribution rate among all the genes enriched in the top 15 functional pathways, which means that targets of Huanglian could play primary therapeutic roles in the treatment of disease, and the target contribution rate of Chen botanical drugs is slightly lower, which indicates that the relatively low target utilization rate may play a role in assisting Jun botanical drug in the treatment process. Zuo botanical drug have the lowest target contribution rate, indicating that the ability of therapeutic role in the treatment of diseases is slightly weak, and may play an auxiliary role in other aspects. The above results once again confirm the major functional role of the Jun botanical drug Huanglian, the auxiliary effects of the Chen botanical drugs Huangqin and Huangbo, and the supplementary effects of the Zuo botanical drug Zhizi in the functional level of HJD.

### C-T-P Network Construction and Analysis

In the process of treating complex diseases, TCM formula usually acts in the form of multi-component and multi-target. These components and targets form the most direct target-protein network, which can reflect some therapeutic effects but cannot reflect the propagation mode of this therapeutic effect. More and more evidences show that the therapeutic effect of drugs on diseases can be propagated through PPI (Andras et al., 2013). Hormozdiari et al. proposed that identified potential multiple-drug targets in pathogenic PPI networks can help us to better discover the therapeutic effect of drugs (Hormozdiari et al., 2010). Chu et al. applied a nonlinear stochastic model and maximum likelihood parameter estimation to identify the cancer-perturbed PPI involved in apoptosis and to identify potential molecular targets for the development of anti-cancer drugs (Chu and Chen, 2008). How to characterize this propagation effect has not been systematically reported.

In this study, we first constructed the C-T network of HJD, then integrated multiple PPI data to construct a comprehensive PPI network. C-T network and PPI networks were integrated as the C-T-P network. By analyzing the C-T-P network, comprehensive information can be obtained and intricate relationships that manage cellular activities can be revealed. In a network, the number of nodes directly interacting with a node is called degree. Several reports have confirmed that the greater the degree, the more biological functions it participates in, and the stronger its biological importance. Under this concept, we made further analysis of C-T-P network. Our results show that the targets of Jun botanical drug Huanglian has the highest average degree 131.75 in C-T-P network. It indicates that these targets affect more proteins in the C-T-P network and have the possibility to play more important roles. By comparative analysis, we found that the degree of huangqin and huanngbo in C-T-P network are 102.46 and 110.33, respectively. The degree of targets of Huangqin and Huangbo are relatively smaller, which indicate that the number of target protein is not as high as that of Jun botanical drug and may play a supplementary role. Zhizi has the lowest average degree 101.42, while indicates that the number of targeted proteins is smallest, and together with Chen botanical drugs to assist the Jun botanical drug.

### Functional Communities Structure Predication and Analysis

In complex life activities such as diseases, development, and drug intervention, etc, a plurality of genes, proteins, and other constituent components in cells are involved, and these genes, proteins, and components form a complex regulation network in cells. In the process of drug intervention, drugs play an intervention regulatory role on the complex network by targeting specific proteins. This intervened regulation and intracellular gene regulation network form a drug-target-pathway complex network at the molecular level. Further research found that the neighbors of drug responding genes in the network tend to be related to the same or similar intervention responses (Li and Zhan, 2006; Zhang et al., 2014).

Genes with the same drug response are often functionally related and form biological network communities (Tari et al., 2005). At the molecular level, the community can be considered as a group of genes, proteins or metabolites that are functionally related, physically interact or jointly response to drug. The molecules in these communities usually jointed together to drive a biological process or respond to the treatment of drugs (Tripathi et al., 2019). For drug intervention in complex diseases, single gene analysis cannot effectively consider the cooperative relationship among genes, and is difficult to explain its biological mechanism. However, community-based analysis can identify response gene sets with cooperative relationships. Revealing the functions of these simple network modules at the molecular level is the key step for understanding the drug response regulation mechanism of more complex networks and even for understanding the mechanism of drug treatment.

**FIGURE 4:**
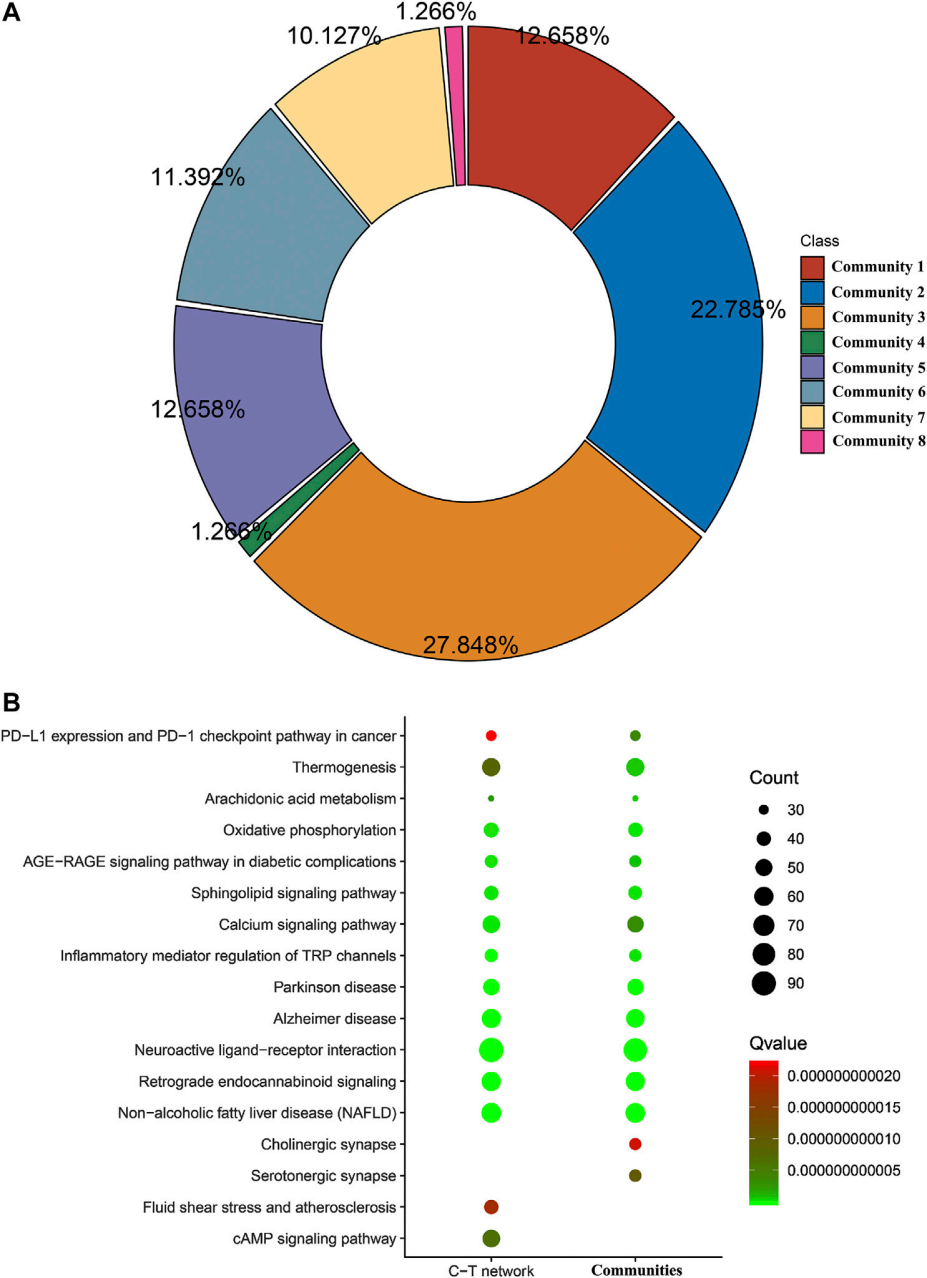
The predicated communities of C-T-P network of HJD **(A)**. Different color represents different communities. The functional similarity analysis between C-T-P network and communities in HJD **(B)**.

**FIGURE 5:**
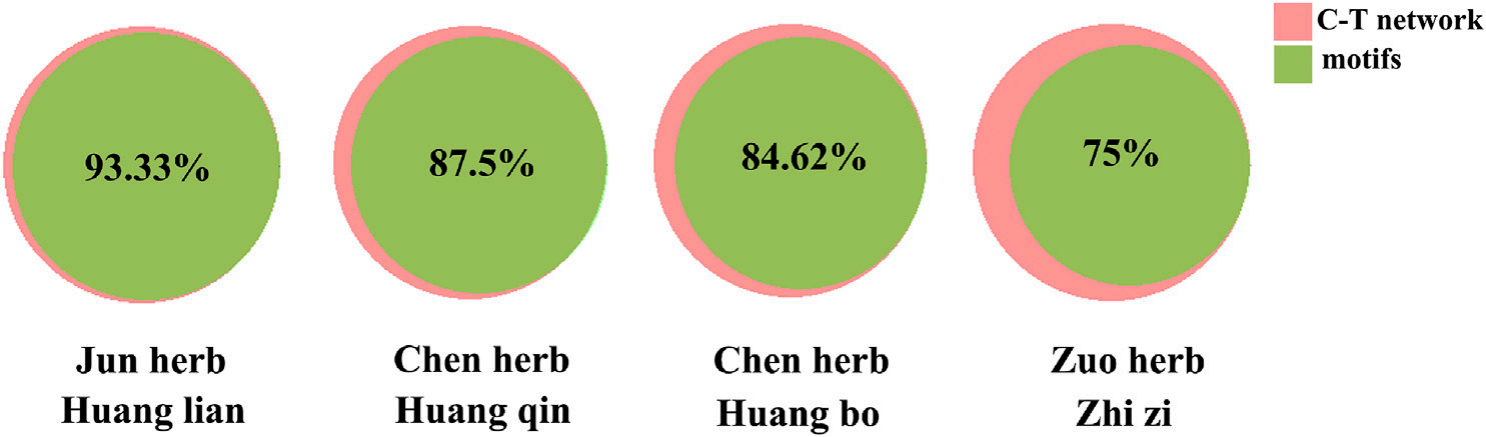
Venn diagram was used to visualize the overlap number between C-T-P network and communities in HJD, the pink represents the C-T-P network, and the green represents communities.

In this study, we construct the C-T-P network by integrating C-T network of HJD and PPI network. This extremely complex C-T-P network with 12,324 nodes and 84,138 interactions is difficult to clarify drug MOA, so discovery of functional communities in the C-T-P network is very important for understanding the organization and function units of the HJD under the concept of the compatibility principle “Jun-Chen-Zuo-Shi”. The extraction of these community structures can reduce the dimension of complex networks and could be considered as the key factor for further clarify the compatibility principle of HJD’s “Jun-Chen-Zuo-Shi”, we identify the functional communities in the C-T-P network based on the information graph algorithm combining random walk theory and huffman encoding. The algorithm performs to optimize the discovery of communities in C-T network heuristically by using a reasonable global metric. The results show that 8 significant functional communities are found in the C-T-P network (Figure 4A). In order to determine whether communities found in HJD can represent their complete C-T network. We evaluate the importance of communities at the gene functional level based on enrichment pathway analysis. The analysis results showed that genes enriched pathways of HJD communities accounts for 93.4% of genes enriched pathways of the full C-T network in HJD (Figure 4B), which indicated that the enriched pathways of genes involved in communities of HJD are highly compatible with enriched pathways of genes in C-T network. Further analysis of the components identified by these functional communities shows that 93.33, 87.5, 84.62, and 75% of the components in the Jun botanical drug Huanglian is covered by functional communities, which once again indicates that Huanglian plays a leading role in the function of C-T-P network (Figure 5).

### Coverage Rate Based on Pathogenic Genes

**FIGURE 6:**
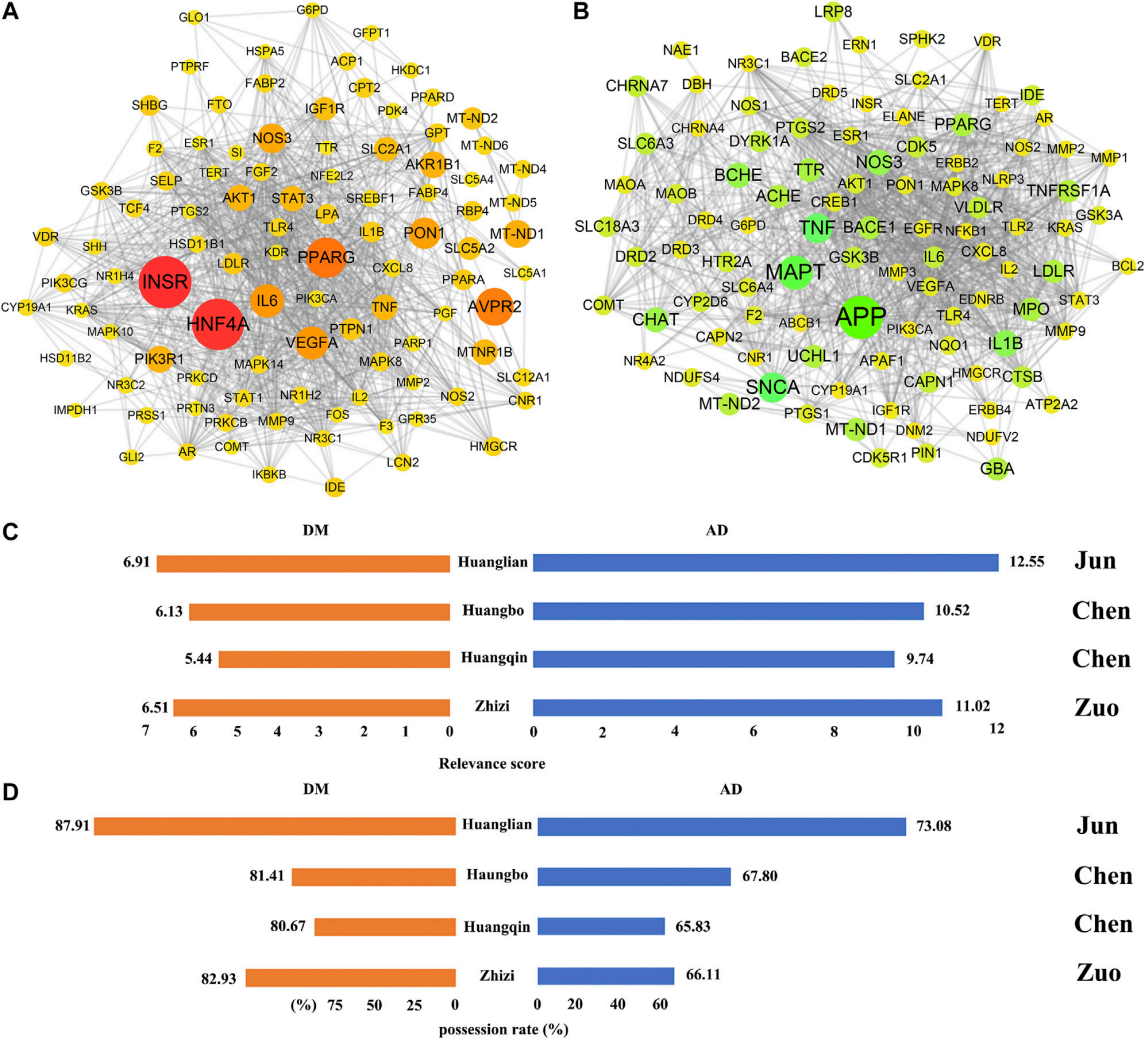
The disease weight gene regulatory network of diabetes mellitus **(A)** and alzheimer’s disease **(B)**. The size and color of the node represents the relevance score of herbal therapeutic targets. The bar chart represents the average relevance score **(C)** and possession rate **(D)** of overlap of botanical drug targets and pathogenic genes.

In order to better explore how HJD exerts its therapeutic effect based on the compatibility principle of “Jun-Chen-Zuo-Shi”, DM and AD were selected for further evaluation. Both of two diseases have been reported with significant therapeutic effects of HJD. Pathogenic genes of both diseases were collected from GeneCards database (Safran et al., 2010). PPI data of both diseases were extracted from STRING database (Szklarczyk et al., 2019). The weighted gene reulatory network of disease was constructed by mapping pathogenic genes to PPI data, the weight was assigned by using relevance scores in GeneCards database (Figure 6A,B; Supplementary Table S1). The relevance score of genes in GeneCards takes into account three aspects: the frequency of the term in the disease related document would raises the score, while the frequency of the term in disease related documents across the site would lower the score, and the size of the subﬁeld containing the term, if the term appears in a smaller ﬁeld, such as gene name, the score would be increased (Stelzer et al., 2016). This indicates that the higher the relevance score, the more important that the genes are involved in pathogenesis of the disease. For further analyzed the “Jun-Chen-Zuo-Shi” compatibility principle of HJD, we design a pipeline to capture the role of each botanical drug in HJD based on botanical drug targets and their associated pathogenic genes. Firstly, we get common gene datasets by overlapping pathogenic genes of each diseases and component targets of each botanical drug, and then analyze the average relevance score of the common gene datasets, then we calculate the possession rate by compare the common gene datasets to targets genes of each botanical drug in HJD. For DM, 87.91% of the targets in the Jun botanical drug Huanglian overlap with the pathogenic genes with an average relevance score of 6.91, 80.67% and 81.41% of the targets in the Chen botanical drugs Huangqin and Huangbo overlap with the pathogenic genes with average relevance scores of 5.44 and 6.13, and 82.93% of the targets in the Zuo botanical drug Zhizi overlap with the pathogenic genes with an average relevance score of 6.51. For AD, 73.08% of the targets in the Jun botanical drug Huanglian overlap with the disease genes with an average relevance score of 12.55, 65.83 and 67.80% of the targets in the Chen botanical drugs Huangqin and Huangbo overlap with the pathogenic genes with average relevance scores of 9.74 and 10.52, and 66.11% of the targets in the Zuo botanical drug Zhizi overlap with the pathogenic genes with an average relevance score of 11.02 (Figure 6). The above results show that the Jun botanical drug Huanglian has the highest possession rate and average relevance score, which indicate that Huanglian acts on as many important targets as possible in pathogenic genes.

### Calculation and Analysis of Propagation Coefficient

The interactions between genes or proteins in cells form complex biological networks. Molecular interactions in biological networks have dynamic and spatiotemporal specific features. At present, protein interaction network and drug regulatory network can only provide static interaction information. In the function analysis of drug targets and pathogenetic genes, the dynamic characteristics of molecular interactions are more significant than static characteristics for understanding the MOA and propagation features of drug intervention in the disease networks. In order to analyze and understand the propagation characteristics of drugs in the disease network more effectively, this study proposed an important monitor which named as propagation coefficient to characterize the drug response network-propagation characteristics by integrating the data of botanical drug targets, PPI, and pathogenic genes. The propagation coefficient contains propagation modes and effector proteins, which could be used to indicate the propagation power of drug. Based on the novel calculate method, the propagation coefficient of single botanical drug in HJD is analyzed, and the scientific basis of the compatibility rule of “Jun-Chen-Zuo-Shi” is revealed from the perspective of propagation characteristics.

**FIGURE 7:**
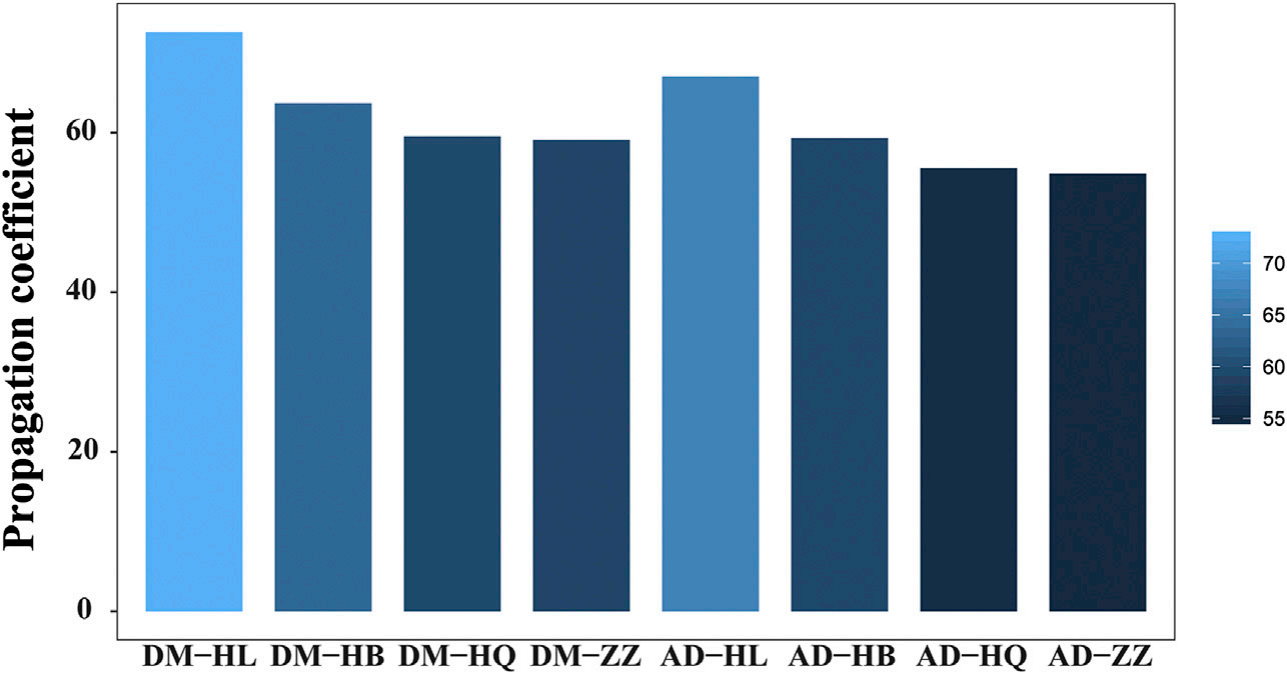
The propagation coefficient of HJD botanical drugs in diabetes mellitus (DM) and alzheimer’s disease (AD).

The propagation coefficient value of each botanical drug in HJD is calculated and showed in Figure 7. According to the calculation results, for DM and AD, the Huanglian with a propagation coefficient of 72.62 and 67.05, the Huangbo with a propagation coefficient of 63.69 and 59.30, the Huangqin with a propagation coefficient of 59.55 and 55.54, and the Zhizi with a propagation coefficient of 59.10 and 54.87, respectively. From the above analysis, Huanglian has highest propagation coefficient both in DM and AD, the propagation coefficient of Haungqin and Huangbo is lower than Huanglian and Zhizi has the lowest propagation coefficient, which indicate that Huanglian plays a major role in disease treatment by spreading the intervention effect at a more powerful level, and Chen botanical drugs and Zuo botanical drugs play a role in assisting Huanglian. This once again confirmed the compatibility rules of “Jun-Chen-Zuo-Shi” in HJD at quantitative level.

### Experimental Evaluation

In order to further explore the accuracy and reliability of the above strategies for analyzing the compatibility rules of HJD “Jun-Chen-Zuo-Shi,” components absorbed into blood were used to validate our strategy. In the study of complex components system of TCM, it is generally believed that the components which can be absorbed into blood are the active components with therapeutic effect. Analysis of the components in blood after oral administration of TCM is an effective and accurate way to study the substance basis of drug effect of TCM. The absorbed components in rat plasma after oral administration of HJD were collected from the previous reports (Zuo et al., 2014). A total of 22 prototype components were obtained, the detailed information was shown in Supplementary Table S2.

For the components obtained by functional communities structure prediction and active components screening in the above strategy, 26.67% of the predicted components in the Jun botanical drug of Huanglian overlap with the components were absorbed into blood; 10.26 and 7.5% of the predicted components in the Chen botanical drugs Huangqin and Huangbo overlap with the components were absorbed into blood. There is no overlap between the predicted components and the components were absorbed into blood in the Zuo botanical drug Zhizi. The above results indicate that the proportion of components in Jun botanical drug were absorbed into blood is higher, followed by Chen botanical drugs, which confirm the accuracy and reliable of our analysis strategy. Meanwhile, the results once again confirm the important role of the Jun botanical drug Huanglian through a higher absorbed into blood rate, the auxiliary effects of the Chen botanical drugs Huangqin and Huangbo, and the supplementary effects of the Zuo botanical drug Zhizi.

## Discussion

TCM usually exerts its efficacy in the form of formula, which is not only a simple combination of botanical drugs, but follows reasonable compatibility principles to treat complex diseases (Sucher, 2013). The main purpose of these compatibility principles is to enhance efficacy or reduce toxicity, so that different chemical components in botanical drugs can promote, coordinate, and restrict each other, thus ensuring the safety and effectiveness of clinical medication (Sucher, 2013). “Jun-Chen-Zuo-Shi” is one of the most common used rules in the compatibility principles of TCM (Yao et al., 2013; Wu et al., 2014). The botanical drugs in a formula can be divided into “Jun”, “Chen”, “Zuo” and “Shi” botanical drugs according to their functions. Based on the compatibility principles of “Jun-Chen-Zuo-Shi”, the function of each botanical drug and its relationship with other botanical drugs are revealed. For example, Yujinfang is based on the compatibility rules of “Jun-Chen-Zuo-Shi” in the treatment of cardiovascular and cerebrovascular diseases (Li et al., 2014). The “Jun” botanical drug *Curcuma wenyujin* Y.H.Chen and C. Ling accounts for the largest proportion of active ingredients and action targets, and treats diseases by acting on the main targets of cardiovascular and cerebrovascular diseases. “Chen” botanical drug *Gardenia jasminoides* J. Ellis can enhance the effect of *Curcuma wenyujin* Y.H.Chen and C. Ling. The “Zuo” and “Shi” botanical drugs can achieve their auxiliary effects by reducing the toxicity of *Curcuma wenyujin* Y.H.Chen and C. Ling and *Gardenia jasminoides* J. Ellis. Many botanical drugs in TCM have both unique effects and strong toxicity in clinical application. According to the needs of clinical treatment, the effectiveness of these botanical drugs should be utilized as much as possible and the toxic and side effects should be reduced at the greatest extent. Based on the compatibility rules of “Jun-Chen-Zuo-Shi”, botanical drugs with toxicity usually are compatible with other botanical drugs to inhibit their toxicity and side effects and to play unique curative effects. This is an important aspect of improving the efficacy of TCM. For example, *Pinellia cordata* N.E.Br. in Xiaobanxia decoction is a commonly used medicine for resolving phlegm and arresting vomiting, but it is toxic. Compatibility with *Zingiber officinale* Roscoe can not only relieve the toxicity of *Pinellia cordata* N.E.Br. but also enhance the anti-vomiting effect of *Pinellia cordata* N.E.Br. to achieve synergistic effect (Gong-Chang et al., 2015).

Recently, more and more attention has been paid to the practice of verifying and explaining the compatibility theory of TCM by using different modern technologies, such as component combination and computer modeling (Wu et al., 2014). However, most of these analysis of compatibility rules of “Jun-Chen-Zuo-Shi” in TCM only focuses on one or several aspects. There is a lack of systematic and multi-dimensional analysis of the compatibility rules of “Jun-Chen-Zuo-Shi.” Network pharmacology mainly focus on problems from the perspective of mutual connection, which is exactly consistent with the core idea of TCM (Li and Zhang, 2013). Therefore, the application of network pharmacology in Chinese medicine research has unique advantages and great development potentiality. However, most of the current network pharmacology research focuses on the interpretation of the functional mechanism of formula in the treatment of specific diseases, and does not interpret the compatibility rules of “Jun-Chen-Zuo-Shi” at a systematic level.

In this study, the compatibility rules and possible mechanisms of TCM in treating complex diseases are analyzed through six detail properties, include the physical and chemical properties of each component in single botanical drug of HJD, ADME properties and functional enrichment analysis of component targets, the characterization of degree and key communities in C-T-P network, the characterization of intervention propagation properties in the treatment of different complex diseases. The pharmacological action of drugs depends on the physical and chemical properties of drugs, which can reflect ADMET characteristics of drugs in the body and is also a basic attribute to be considered in interpreting the compatibility rules of TCM. Complex networks are made up of a large number of nodes, of which the important nodes are a few special parts that can deeply influence on the structure and function of the whole network. Node degree in the topological structure can reflect the importance of nodes, and is a key topological parameter to characterize the most influential nodes in the network (Lv et al., 2014). For example, in order to evaluate the influence of components in ZZW on FD treatment, Wang et al. constructed a contribution index model based on the topological parameter degree in the network. By using this algorithm, they selected key component groups for FD treatment and clarified possible cooperative mechanisms (Wang et al., 2018).

Modularity is a very important characteristic of complex networks and a common phenomenon in biological systems (Yang and Leskovec, 2012). Studying the response network modules of different chemical components in different botanical drugs is very important to analyze the drug mechanism. It is also an important way to systematically validate the rules of “Jun-Chen-Zuo-Shi.” The intervention effect of compounds in herbal medicine could propagate through PPI (Comola and Prina, 2013). The propagation of this intervention in the network has specific propagation modes and paths. The propagation coefficient defined based on the propagation method and path is also different in the principle of “Jun-Chen-Zuo-Shi.” Enrichment analysis of gene function is a routine method for gene group function analysis, which is of great significance for revealing the molecular mechanism of different Chinese medicines in formula, and is a further explanation for the correlation between compatibility rules and mechanisms of Chinese medicines based on “Jun-Chen-Zuo-Shi”. Based on the above-mentioned features, we designed a new system pharmacology strategy, which can systematically interpret the compatibility rule of “Jun-Chen-Zuo-Shi” from structure to function and then to propagation mode at a multi-level perspective.

Taking HJD as an example, we deeply decoded the compatibility rules of TCM. Jun botanical drugs play a leading role in formula, and exert the strongest effect in treating diseases through the best chemical properties, the highest occupancy rate of active components, the highest topological structure of drug action network, the highest occupancy rate of functional communities of drug response network and the highest drug intervention coefficient and potential molecular pathways of action. The Chen botanical drugs can enhance the pharmacological effects of Jun botanical drugs and reduce the dosage required by Jun botanical drugs through slightly lower functional targets. Zuo and Shi botanical drugs could be improved the bioavailability and active component of Jun and Chen botanical drugs. In addition, the C-T-P network proves the multidirectional pharmacological treatment mechanism of TCM, i.e. multiple components, multiple targets and multiple therapeutic effects. The prescription principle of different botanical drugs provides a unique opportunity to explore multiple therapeutic mechanisms according to the efficacy of botanical drugs. The combination of different botanical drugs can not only treat diseases by increasing bioavailability or promoting the synergistic effect of different botanical drugs, but also reduce the toxicity of some botanical drugs. The synergistic mechanism and toxicity-reduced effect embodied by these compatibility rules also indicate that the botanical drug combination is more effective than the use of single botanical drug.

Additionally, HJD was widely used in the treatment of AD and PD, thus, the study of brain tissue distribution of HJD is particularly important. Passing through the blood-brain barrier (BBB) is crucial for drugs to enter the central nervous system and play therapeutic roles. Through the analysis of components absorbed into blood in HJD, we found the components that can be absorbed into blood in the Jun botanical drug of Huanglian have strong permeability of BBB (>0.3), including berberine, palmatine, coptisine, and epiberberine. However, specific components wogonin and oroxylin a absorbed into blood in the Chen botanical drug of Huangqin only have moderate permeability of BBB. This proves once again that Jun botanical drugs are the core of the formulas, and they play a major role in treating diseases.

In this study, the compatibility rule of “Jun-Chen-Zuo-Shi” of HJD was in-depth decoded from multi-scale perspective. At the components and targets level, the Jun botanical drug huanglian plays a leading role at the functional level and has good performance in most physical and chemical properties, ADME properties and functional enrichment analysis of component targets. At the C-T-P interaction level, the leading role of the Jun botanical drug huanglian is also confirmed by having the highest average degree in C-T-P network and targets coverage rate of functional communities. At the intervention propagation level, the Jun botanical drug Huanglian has highest propagation coefficient both in DM and AD, this once again confirmed the Jun botanical drug plays a leading role in a formula for treating diseases. Finally, the results of experimental validation showed that the proportion of components in Jun botanical drug were absorbed into blood is higher than Chen and Zuo botanical drugs, including berberine, palmatine, and coptisine etc. Our approach confirmed the compatibility rules of “Jun-Chen-Zuo-Shi” in HJD at multiple quantitative level. This research shows the scientific basis of “Jun-Chen-Zuo-Shi” from a multi-dimensional perspective, which providing a good methodological reference for the subsequent interpretation of key components and speculation mechanism of the formula.

 However, there are still some limitations of this study. This research is a computational pharmacological work based on pharmacological experiment data and public data. Pharmacological calculation is the forerunner and basis of the experiment, which provides a feasible scheme to reduce the verification scale for the experiment. Evidence from pharmacological experiments should be added in future research.

## Data Availability Statement

The raw data supporting the conclusions of this article will be made available by the authors, without undue reservation, to any qualified researcher.

## Author Contributions

K-XW and YG contributed equally to this work. D-GG, A-PL, and X-MQ provided the concept and designed the study. K-W and YG conducted the analyses. K-XW and YG wrote the manuscript. K-XW, YG, W-XG, X-FY, CW, L-YF, and X-FG participated in data analysis. X-MQ, G-HD, LG, and A-PL provided oversight. D-GG and A-PL contributed to revising and proof-reading the manuscript.

## Funding

This study is financially supported by the Startup fund from Southern Medical University (grant No. G619280010), the Natural Science Foundation Council of China (grant No. 31501080), Hong Kong Baptist University Strategic Development Fund (grant No. SDF13-1209-P01, SDF15-0324-P02(b) and SDF19-0402-P02), the Key Laboratory of Effective Substances Research and Utilization in TCM of Shanxi Province (No. 201705D111008-21), Hong Kong Baptist University Interdisciplinary Research Matching Scheme (grant No. RC/IRCs/17-18/04), the General Research Fund of Hong Kong Research Grants Council (grant No. 12101018, 12100719, 12102518).

## Conflict of Interest

The authors declare that the research was conducted in the absence of any commercial or financial relationships that could be construed as a potential conflict of interest.
