# Supplementary material for: A Novel Strategy for Decoding and Validating the Combination Principles of Huanglian Jiedu Decoction From Multi-Scale Perspective
Source: Front Pharmacol. 2020 Dec 4;11:567088. doi: 10.3389/fphar.2020.567088 (PMC7789881; doi:10.3389/fphar.2020.567088)
Supplement: Supplementary file 2 [file table2.docx]

Table S2 The detailed information on components absorbed into blood in HJD.

| NO. | Formula | Component |
| --- | --- | --- |
| 1 | C_16_H_21_O_10_ | Geniposidic acid |
| 2 | C_17_H_24_NaO_11_ | Gardenoside |
| 3 | C_17_H_24_NaO_11_ | Scandoside methyl ester |
| 4 | C_20_H_24_NO_4_ | Phellodendrine |
| 5 | C_20_H_24_NO_4_ | Magnolflorine |
| 6 | C_23_H_34_NaO_15_ | Genipin-1-gentiobioside |
| 7 | C_17_H_24_ NaO_10_ | Geniposide |
| 8 | C_19_H_14_NO_4_ | Coptisine |
| 9 | C_20_H_18_NO_4_ | Epiberberine |
| 10 | C_20_H_20_NO_4_ | Jateorhizine |
| 11 | C_20_H_18_NO_4_ | Berberine |
| 12 | C_21_H_22_NO_4_ | Palmatine |
| 13 | C_21_H_19_O_11_ | Baicalin |
| 14 | C_22_H_21_O_11_ | Dihydrooroxylina-7-O-glucuronide |
| 15 | C_21_H_18_NaO_11_ | Norwogonin-7-O-glucuronide |
| 16 | C_22_H_21_O_11_ | The isomer of dihydrooroxylina-7-O-glucuronide |
| 17 | C_22_H_21_O_11_ | Oroxylin A 7-O-glucuronide |
| 18 | C_22_H_21_O_11_ | Wogonoside |
| 19 | C_21_H_19_O_11_ | Norwogonin-8-O-glucuronide |
| 20 | C_23_H_23_O_12_ | 5, 7-Dihyroxy-6, 8-dimethoxy flavone-7-O-glucuronide |
| 21 | C_16_H_13_O_5_ | Wogonin |
| 22 | C_16_H_13_O_5_ | Oroxylin A |
